# Proceedings of the Lippe Society

**Published:** 1889-01

**Authors:** Geo. H. Clark

**Affiliations:** Secretary


### PROCEEDINGS OF THE LIPPE SOCIETY.

The 127th meeting of the Lippe Society was held on Tuesday evening, December 11th, 1888. After the minutes of the preceding meeting were read and approved, Dr. C. Carleton Smith read an instructive paper on "Sticta Pulmonaria," illustrating its action in a case of nasal catarrh. The paper will be published in this journal. Dr. Farley then said as far as he had observed, the nasal discharge characteristic of Sticta dries up very speedily. In reply to Dr. Lee, Dr. Farley said that the remedies having stopped feeling at root of nose, worse lying down at night are, Amm. carb., Lycop., and Puls. Amm. carb., child awakens from sleep crying for want of ability to breathe through nose. Lycop., he awakes rubbing the nose, because of the stoppage.

Dr. Lee related the case of a child in which the annual attack of hay-fever had been kept off by having a sliced onion on the mantel in the room in which the child slept. Previous to this both the child and his mother had an attack of hay-fever unless they crossed the ocean. Dr. Lee also mentioned a case in which a lady took several doses of Acon. to check a cold. She succeeded in checking the cold, and she also succeeded in getting a proving of Aconite.

Dr. Farley had found that Allium cepa will rapidly cure cold that begins with the usual violent nasal and conjunctival symptoms of that remedy: fluid coryza, bland discharge. Dr. Clark spoke of a case of tertiary syphilis, in which the characteristic of Sticta: constant desire to blow nose, without discharge, was present. Sticta cured not only that symptom but others that were present.

Dr. James then read a paper entitled, "Metastasis of Disease." The paper being opened to discussion, Dr. Lee said that there could be no question that what the allopathists usually term the sequelæ of disease, are nothing but metastases. He then spoke of his own case. Fifteen years ago he had an attack of ophthalmitis of the right eye. The inflammation was severe; there was great photophobia; much pain, and lids were agglutinated in morning. Knowing nothing of Homœopathy, he was advised to apply Croton oil back of the ears. The inflammation disappeared in three days. He never discovered the bad effects until some years after, when he found vision of right eye almost nil. The practical question is, can we do anything for such cases? That is, cases in which disease has been suppressed, and some chronic affection arises. The experience of all present goes to show that Hahnemannian Homœopathy can, after a time, turn aside the ill effects of badly treated disease.

Every member gave testimony to the effect that the worst, and most troublesome cases met with are uterine affections, in which modern gynecologists have been tampering with the uterus by making various applications. Horrible suffering, and frequently incurable mania often result from such treatment. Dr. Lee said he knew of a lady who was now an inmate of an asylum, who had ligatures applied to hemorrhoids, previous to which her health had been good.

In gynecological cases the cautery was less powerful for harm than drugs; as is also the knife.

Dr. James spoke of a lady who had been under Dr. Lippe's care. Before she knew of Homœopathy, she had been in the hands of the regulars (?). Her old-school physician said her troubles were due to ulceration of the lining membrane of the uterus, and that the mouth of the uterus would have to be dilated. He introduced several sponge-tents for this purpose, and, after having his patient suffer much agony, he found nothing. The lady then went abroad and was under the treatment of a celebrated London gynecologist, who said she had ulceration of the neck of the womb, and who applied Nitrate of Silver, and converted the neck into a callous mass. After this she went to Paris, where the neck was amputated. On her return to this country she applied to Dr. Lippe for treatment, and afterward

Dr. James treated her, together with Dr. Lippe. Dr. James had never seen a more terrible case. After many remedies, which gave only temporary relief, she finally developed lung trouble, from which she died. One of her most prominent symptoms, and which was present in an aggravated form, was great goneness in region of stomach. Stannum and Phos. were the only remedies which controlled this symptom, after Sepia had failed. The remedies were given simply according to indications.

Dr. Clark stated that he had found, in cases coming from old-school hands where goneness of the stomach was present, Digitalis had been given.

Dr. Farley then presented the following case: Mrs. J. Y., æt. forty, married, dark and spare. Has suffered for twenty years with palpitation, pain, dyspnœa, and anxiety in cardiac region. She has "suffered much from much doctoring." On examination the following symptoms are elicited:—Smothered feeling in cardiac region, with terrible anguish and fear of death; this is produced or aggravated by the least hurry, excitement, unexpected noise, or on being suddenly spoken to. Sensation of a huge hand grasping chest in cardiac region. Rapid, forcible beating of heart, felt throughout the body; worse lying on left side; excluding from crowds and from friendly callers. Weak, trembling feeling for days after a severe attack. Pain goes through to left shoulder and down arm.

Perturbed state of mind, *can't help thinking of her state, and deems it hopeless.* Worse from thinking of it; better if she can keep her mind occupied from self. Continual dread of immediate death, and also of death in next attack. Sense of shock, like from fright, in cardiac region, and in solar plexus. May feel perfectly well for a short time—an hour or two—then, suddenly, without warning or provocation, or from emotion, will feel at her worst. Sleeps well, and when first opening the eyes feels perfectly well, but just as soon as she is fully awake and collected mentally, the awful gloom creeps over her like a cloud.

Sinking in solar plexus and cardiac region. Waves of heat going from nape of neck to forehead. Weakness and dejection after coitus; absence of all desire. Cramping, drawing pain in right ovarian region, better from doubling up or from hard pressure; must sit down. Weak, all-gone feeling in abdomen, must wear a bandage to support abdomen.

Agnus cast., and Lycop. were suggested for study in connection with this case.

After suggestions from several members as to the order of papers to be presented, the society adjourned.

GEO. H. CLARK, *Secretary.*

